# No impacts of glyphosate or *Crithidia bombi*, or their combination, on the bumblebee microbiome

**DOI:** 10.1038/s41598-023-35304-3

**Published:** 2023-06-02

**Authors:** Edward A. Straw, Robin Mesnage, Mark J. F. Brown, Michael N. Antoniou

**Affiliations:** 1 Department of Botany, Trinity College Dublin, Dublin, Ireland; 2 Buchinger Wilhelmi Clinic, Wilhelmi-Beck-Straße 27, 88662 Überlingen, Germany; 3 Centre for Ecology, Evolution and Behaviour, Department of Biological Sciences, School of Life Sciences and the Environment, Royal Holloway University of London, Egham, UK; 4 Gene Expression and Therapy Group, King’s College London, Faculty of Life Sciences and Medicine, Department of Medical and Molecular Genetics, Guy’s Hospital, London, SE1 9RT UK

**Keywords:** Microbial communities, Entomology, Environmental impact

## Abstract

Pesticides are recognised as a key threat to pollinators, impacting their health in many ways. One route through which pesticides can affect pollinators like bumblebees is through the gut microbiome, with knock-on effects on their immune system and parasite resistance. We tested the impacts of a high acute oral dose of glyphosate on the gut microbiome of the buff tailed bumblebee (*Bombus terrestris*), and glyphosate’s interaction with the gut parasite (*Crithidia bombi*). We used a fully crossed design measuring bee mortality, parasite intensity and the bacterial composition in the gut microbiome estimated from the relative abundance of 16S rRNA amplicons. We found no impact of either glyphosate, *C. bombi*, or their combination on any metric, including bacterial composition. This result differs from studies on honeybees, which have consistently found an impact of glyphosate on gut bacterial composition. This is potentially explained by the use of an acute exposure, rather than a chronic exposure, and the difference in test species. Since *A. mellifera* is used as a model species to represent pollinators more broadly in risk assessment, our results highlight that caution is needed in extrapolating gut microbiome results from *A. mellifera* to other bee species.

## Introduction

Bees are vital components of ecosystems, providing the majority of animal pollination globally^1^. To humans, bees are important for the products we extract from them (honey and wax etc.), but more significantly, for the pollination they perform, worth $235–577 billion to agriculture each year^2^. There is huge diversity among bees^3^, but one group that are particularly important pollinators of crops are bumblebees^4^. Reflecting global patterns^5^, of the 68 species in Europe, 45% are in decline and 23% listed as vulnerable or worse^6^. Consequently, there is mounting concern over the continued provisioning of ecosystem services by bumblebees. Many factors threaten bumblebee populations, and one wholly anthropogenic factor is pesticide use, which has been linked to harm to wild bees^7,8^.

Pesticides are agrochemicals used to protect crops from diseases and damage. The term pesticide covers a broad array of chemicals, including herbicides, fungicides, insecticides and nematicides among others. Of all pesticides, fungicides are the most diverse and widely used^9^. However, because the fungicide market is so diverse it is the herbicide active ingredient glyphosate that is the most widely used single pesticide globally^10,11^. Herbicides are used to control weeds in crop fields, as well as having a diverse array of other functions in agriculture such as pre-harvest desiccation and for clearing fields for re-seeding^12^. They are also used outside of agriculture, in amenity settings around urban environments or parks, in forests and by consumers. The impacts of herbicides on bees are comparatively under-researched relative to other pesticide types like insecticides^13,14^.

Glyphosate is the best understood and intensely researched herbicide, having been investigated in the context of both human and bee health, with its toxicity being a major topic of debate^15^. For humans, glyphosate was classified as “probably carcinogenic to humans” (Group 2A) in 2015 by the International Agency for Research on Cancer^16^, although this has been questioned by regulatory agencies. For bees, there is contention over whether glyphosate causes mortality in unstressed bees (see^17^ and the response^18^), with the more reliable evidence pointing to this being unlikely^19–21^. However, lethal effects, while important, are not the only impact a pesticide can have on bees^22^. Glyphosate has been found to impact thermoregulation in bumblebees^23^, and potentially learning capabilities^24^. Whether these effects occur at environmentally realistic concentrations or conditions remains unclear, and there is a lack of large-scale semi-field, or full-field work on the topic.

Glyphosate exerts its herbicidal action by inhibiting the shikimate pathway found in plants, causing a lack of aromatic amino acid biosynthesis^11^, which bees do not possess. However, some microorganisms (bacteria, fungi) share this pathway, and it has been demonstrated that glyphosate and glyphosate-based herbicide formulations do impact the shikimate pathways in the gut of animals^25^. Some studies have suggested that glyphosate can also inhibit the shikimate pathway in microorganisms that are present in the bee gut^26–29^. Accordingly, research has looked into whether the bee microbiome can explain the negative impacts of glyphosate. This is plausible as the bee microbiome plays a crucial role in digestion as well as protection of the bee from gut parasitism^30^, so changes to it could compromise bee health. Several publications have found that in honeybees, chronic exposure to reportedly environmentally relevant concentrations of glyphosate can cause changes in the microbiome^26–29^. This was linked to immune dysregulation and a decreased resistance to colonisation by opportunistic pathogens^26^.

A key finding on the impact of glyphosate on the honeybee microbiome is the interaction of the altered microbiome with parasites. When honeybees were fed 10 mg/L of glyphosate in sucrose for 5 days, there was no increase in mortality^26^. Similarly, exposure to a representative infection with *Serratia marcescens* alone caused only a low level of mortality. However, in combination with glyphosate the mortality reached ~ 90%, demonstrating that stressors synergise to cause more mortality then either alone. This synergistic mortality was directly attributed to the changes in the microbiome composition caused by glyphosate. This work has since been confirmed and further developed^27–29,31^.

*Serratia* has only been found in bumblebees exhibiting dysbiosis^32^ but these bees are host to a range of other parasites. One of the most ubiquitous is *Crithidia bombi*, a trypanosome gut parasite^33–38^. Infection with *C. bombi* in an otherwise unstressed bee is not benign but will not cause serious mortality or impairment to fitness^21,33,39–41^. However, alongside other stressors it can additively or synergistically cause serious reductions in the bee’s overall fitness^39,42,43^. However, emerging evidence suggests that *C. bombi* exhibits weak synergism potential with pesticides, with no substantive impacts on the whole organism found alongside the insecticide sulfoxaflor^44^. In one study, *C. bombi* was tested alongside glyphosate and no interaction was found with either acute or chronic exposure on mortality, sucrose consumption, weight change or parasite intensity^21^ . However, it is possible that if a combination of glyphosate and *C. bombi* alter the gut microbiome, other untested metrices of bee health, such as reproduction or hibernation success may be impacted.

Recently, three studies have found limited, and at times somewhat conflicting, results of chronic glyphosate exposure in bumblebees, using both glyphosate as an active ingredient and glyphosate-based formulations^29,45,46^. In *Bombus impatiens*, both glyphosate and the formulation were found to impact microbiome composition, notably *Snodgrassella* abundance^29^. In *Bombus terrestris*, both glyphosate and the formulation altered cellular and physiological processes, including processes associated with oxidative stress regulation and cellular adhesion^45,46^. Further, either highly limited or no impacts on gut bacterial composition were observed^45,46^, although some impacts on fungal species were observed. Notably, no study found impacts on mortality or sucrose consumption at field realistic chronic exposure rates. Where these experiments found impacts, the exposures used were marginally above field realistic glyphosate residue levels given the length of exposure (5 to 10 days)^19^.

Here we build on the established honeybee literature and emerging bumblebee literature by reporting for the first time the impact of acute glyphosate exposure on the bumblebee microbiome. We used an acute oral exposure to a high dose of glyphosate to test if a worst-case exposure has any impact on the gut microbiome. Additionally, we tested the impact of *C. bombi* on the microbiome, as well as its interaction with glyphosate.

## Methods

### Bees

Three colonies of *Bombus terrestris audax* (Agralan Ltd, Swindon, UK) were maintained on *ad libitum* sucrose and honey bee collected pollen (Thorne, Windsor, UK and Agralan Ltd, Swindon, UK respectively). A total of 10 workers per colony were removed and their faeces screened under 400 × magnification for micro-parasites^35^. No infections were detected.

### Modified OECD 247

The methods employed were modifications of OECD 247^47^ and were partially adapted from^48^. The method was reported in detail in^21^. OECD 247 is an internationally agreed-upon toxicity testing protocol for assessing the effects of acute exposure to an oral solution in bumblebees; see Table 1 for the doses of pesticide and parasite used in the experiment, as well as the sample sizes.

**Table 1:** Treatment groups, pesticide doses and parasite loads.

Control	*Crithidia* only	Positive Control-Dimethoate	Glyphosate only	Glyphosate and *Crithidia*
		4µg	200 μg	200 μg
	10,000 cells			10,000 cells
n = 40	n = 40	n = 40	n = 40	n = 40

Workers were removed from colonies using forceps and transferred to pre-weighed Nicot® cages. Age of bees was not controlled for. The bee and cage were then weighed, and the weight of the bee calculated as the difference between the two measurements. Any bees weighing less than 0.1 g or more than 0.4 g were excluded from the experiment due to their size, a more formal approach to the OECD 247 guidance. Each bee was given an individual ID and was allocated to treatment groups by rank weight order within a colony, which prevents bee weight and colony from confounding the results.

Syringes with clipped tips filled with 50% (w/w) sucrose were added to the Nicot® cages for sustenance. The subsequent day, following the OECD 247 protocol, bees were exposed to the parasite treatments. The parasite inoculum was prepared by removing 40 worker bees from *C. bombi* infected colonies and inducing them to defecate using gentle agitation. The faeces were then purified following^49^. Purified *C. bombi* solution was then diluted to 500 cells per µL in distilled water and mixed 1:1 with 50% (w/w) sucrose to produce the test solution. A 40 µL droplet was fed to the bees, giving a dose of 10,000 cells per bee. Prior work has demonstrated that this method leads to very high infection rates (> 95%) and an environmentally realistic exposure^50^. Control bees were fed with a sham inoculum made of 1:1 distilled water and 50% (w/w) sucrose.

Syringes were removed from the cages for 2–4 h, starving the bees. Then, 40  µL of the parasite solution (or sham solution) was pipetted into a fresh syringe, and this was added to each cage, with the bees then left to feed on this for a further 4 h. The syringe was then removed and consumption verified visually. Bees were returned to ad libitum sucrose with a new syringe of 50% (w/w) sucrose and had a small ball of pollen added (~ 1 g) for a week for the parasite infection to develop, at which point they entered the pesticide exposure period.

### Pesticides

Pesticides were applied as pure active ingredient, glyphosate (Sigma-Aldrich) CAS-no: 1071-83-6 and dimethoate (Sigma-Aldrich) CAS-no: 6-51-5. Dimethoate was used as a positive control, to demonstrate that mortality could be achieved within the experimental set up.

The above steps for exposure were repeated with pesticide-laced treatment solutions replacing parasite solutions. After exposure to the pesticide, mortality was recorded at 0 h, 4 h, 24 h and 48 h. The experiment was conducted in two batches, staggered by a single day to allow for the time-intensive parasite counts to occur. Syringes with sucrose were weighed before being connected to the Nicot cages and again at the end of the experiment (or upon bee mortality) to calculate sucrose consumption.

A 200 µg dose of glyphosate was chosen as the regulatory standard dose for a limit test. It represents essentially the worst-case acute exposure possible, meaning if no impacts are seen then it is highly unlikely an effect occurs under more realistic conditions. A 4 µg dose of a potent insecticide, dimethoate, was used as a positive control, as per OECD 247^47^.

### Parasite intensity

At the end of the 48-h exposure period, bees were moved from Nicot cages to specimen tubes and induced to defecate with gentle agitation. A 10µL micro-capillary tube was used to draw up faeces, which were then transferred to a Neubauer haemocytometer, where parasite intensity was counted (faecal intensity is strongly correlated with whole gut infection intensity as measured by qPCR;^51^). Bees were then placed on ice to chill them, weighed once they were no longer active, placed in 2 mL microcentrifuge tubes and transferred to a − 20 °C freezer. The bees’ end weight enables a change in weight post exposure measurement to be made.

### Gut preparation for microbiome analysis

We followed the protocol of^52^ with modifications. In batches of two, bees were removed from the − 20 °C freezer and one allowed to defrost, with the second placed on ice to delay this process. With the aid of two 100 µL droplets of 0.8% Ringers solution, the whole gut was dissected out, including the honey crop and rectum. This was then immersed in 0.5 mL of 70% ethanol 30% water for 2 s to sterilise (with agitation), and then washed in 0.5 mL distilled water for a further 2 s. All sterilising and washing liquid was single use. The gut was then placed in a microcentrifuge tube and stored on ice until both bees were dissected, at which time samples were returned to − 20 °C. After all dissections were performed, the bees were moved to − 80 °C then shipped to Kings College London on dry ice, where they were returned to − 80 °C. As the sample size for an ecotoxicology trial is larger than that on which it is feasible to perform a gut microbiome analysis, only 15 samples per treatment were assessed for this matrix. Samples were chosen and ordered such that colony and weight could not become confounding because of the sub-sampling. All microbiome work used bees from the first batch.

### 16S rRNA sequencing

The 16s rRNA sequencing was conducted blind to the treatment identities, with alphabetical codes assigned to the treatments. The blind was only dropped after the data were analysed. DNA extraction from bee gut samples was performed using the Zymo Quick-DNA microprep kit (Cambridge Bioscience, Cambridge, UK) following the manufacturer’s instructions. DNA extractions failed for 4 out of the 60 bumblebees included in this study. Polymerase chain reaction (PCR) was performed using the Roche High-Fidelity PCR System (Roche Life Science, Welwyn Garden City, UK). A total of 5 ng DNA was amplified in a reaction volume of 10 µL. The primers for the amplification of the 16S V3-V4 region were: ACACTGACGACATGGTTCTACACCTACGGGNGGCWGCAG (forward) and TACGGTAGCAGAGACTTGGTCTGACTACHVGGGTATCTAATCC (reverse). The PCR reaction mixture included 1 µl of 10X FastStart High Fidelity Reaction buffer, 0.1 µl of 10 µM forward or reverse primers, 1.8 µL of 25 mM MgCl_2_, 0.5 µL dimethyl sulfoxide (DMSO), 0.2 µL of 10 mM PCR Grade Nucleotide Mix, 0.1 µL of 5U/µL FastStart High Fidelity Enzyme Blend and 5.2 µL of nuclease-free water. This was added to 1 µL of diluted samples containing 5 ng DNA and amplified for 35 cycles at 95 °C for 30 s, 55 °C for 30 s, 72 °C for 30 s, and a final extension at 72 °C for 5 min. The size of amplified products was verified by electrophoresis on a 2% agarose gel. We then used 1 µL of a 100 times diluted PCR product in 10 mM Tris/HCl, 1 mM EDTA pH 7.5 buffer in a second round of PCR to add TSP FLD barcodes and Illumina adaptors onto amplification products. The barcoding reaction mix included 1 µL of 10X FastStart High Fidelity Reaction buffer, 1.8µL of 25 mM MgCl_2_, 0.5 µL of DMSO, 0.2 µL of 10 mM PCR Grade Nucleotide Mix, 0.1 µL of 5U/µL FastStart High Fidelity Enzyme Blend and 3.4 µL of nuclease-free water. This was added to 2 μL of Fluidigm Barcode and 1 μL of the 1:100 harvested PCR product. The amplification was done for 15 cycles at 95 °C for 15 s, 60 °C for 30 s, 72 °C for 60 s, and a final extension at 72 °C for 3 min. Barcode attachment was controlled using the Tapestation D1000 instrument (Agilent, CA, USA). An equal volume of each barcoded PCR product was pooled, and the final mixture diluted to 4 nM. The pooled library was loaded onto a 300bpx2 paired-end MiSeq (Illumina, CA, USA), as per the manufacturer’s instructions.

### Whole organism statistics

Statistical analyses were carried out in ‘R’ programming software version 4.2.0^53^. ‘lme4’ version 1.1–29 was used for linear mixed effects models^54^. Parameter estimates and 95% confidence intervals are reported. Confidence intervals crossing zero indicate no significant effect, so a confidence interval of − 1.00 to 1.00 would not be significant, but a confidence interval of − 2.00 to − 1.00 would be significant. Model assumptions were checked graphically. Throughout, the co-variate of batch caused modelling fitting issues and was dropped.

No statistics were conducted for the mortality data (see the results for details). Sucrose consumption and weight change were analysed with a generalised linear model as mixed effects modelling caused fitting issues; this used the model (Metric ~ Treatment + Colony). Weight change was square root transformed to normalise the data. Parasite intensity was analysed with a linear mixed effects model (Parasite Intensity ~ Treatment + (1|Colony)).

### Gut microbiome statistics

The DADA2 pipeline (v 1.16) was used to quantify amplicon sequence variants (ASV) using R v4.0.0^53^. The taxonomy was assigned using the native implementation of the naive Bayesian classifier method from DADA2 with the SiLVA ribosomal RNA gene database v138 for the 16S reads^55^. Cleaned read counts, ASV taxonomies, and the metadata were then combined for an analysis with the phyloseq package v1.32.0^56^. Alpha diversity (mean bacterial diversity in a sample) was estimated by calculating the Shannon index with phyloseq. Statistical significance for alpha diversity was determined with ANOVA tests (Shannon index ~ Treatment + (1|Colony)) followed by Tukey post-hoc tests for multiple comparisons. Microbiome beta diversity (degree of microbial composition similarities between samples) was compared between each sample using non-metric multi-dimensional scaling (NMDS) plots of Bray–Curtis dissimilarities, with the statistical significance of sample clustering evaluated with a permutational ANOVA (PERMANOVA) analysis on the Bray–Curtis dissimilarities with adonis from vegan v2.4-2^57^ (Beta diversity ~ Treatment + (1|Colony)). Differences in microbiome composition were evaluated by determining multivariable association using MaAsLin2 v0.99.12 in R^58^. The statistical significance was corrected for multiple comparisons and presented as False Discovery Rate (FDR).

## Results

### Whole organism results

#### Mortality

All bees in the positive control died, demonstrating the test can detect lethal effects. In all other treatments, there was only one mortality, which was in the *Crithidia* treatment group, making further mortality analysis of no value. As all bees in the dimethoate treatment died within 4 h, this treatment is excluded from all statistical tests below.

#### Sucrose consumption

All bees consumed a similar amount of sucrose, around 0.6 g over the 48-h period (Control = 0.633 g, *Crithidia* = 0.609 g, Glyphosate = 0.622 g, and Glyphosate + *Crithidia* = 0.578 g). There was no impact of treatment on sucrose consumption (*Crithidia*: parameter estimate (PE) = − 0.02, 95% Confidence Intervals (CI) [− 0.09 to 0.05]; Glyphosate: PE = − 0.01, 95% CI [− 0.08 to 0.06]; Glyphosate + *Crithidia*: PE = − 0.05, 95% CI [− 0.13 to 0.02]). There was also no impact of colony (see the Supplementary Materials).

#### Weight change

Bees had similar changes in weight, with only marginal gains in all treatments bar exposure to *Crithidia*, where a small loss was recorded (Control =  + 2.7 mg, *Crithidia* = − 1.9 mg, Glyphosate =  + 2.7 mg, and Glyphosate + *Crithidia* = 3.1 mg). There was no impact of treatment on weight change (*Crithidia*: parameter estimate (PE) =  − 0.04, 95% Confidence Intervals (CI) [− 0.09 to 0.02]; Glyphosate: PE = − 0.03, 95% CI [− 0.10 to 0.04]; Glyphosate + *Crithidia*: PE = − 0.02, 95% CI [− 0.09 to 0.04]). There was also no impact of colony (see the Supplementary Materials).

#### Parasite intensity

Glyphosate did not induce an increase in parasite intensity, and in fact this treatment induced a marginally lower intensity (*Crithidia* = 26,969 cells per µL and Glyphosate + *Crithidia* = 22,356 cells per µL). Overall there was no statistically significant impact of glyphosate on parasite intensity (Glyphosate + *Crithidia*: parameter estimate (PE) = − 4743, 95% Confidence Intervals (CI) [− 10,286 to 970]). As colony was a random effect, its effects are included within the model and not a reportable variable.

### Gut microbiome composition

**Figure 1:**
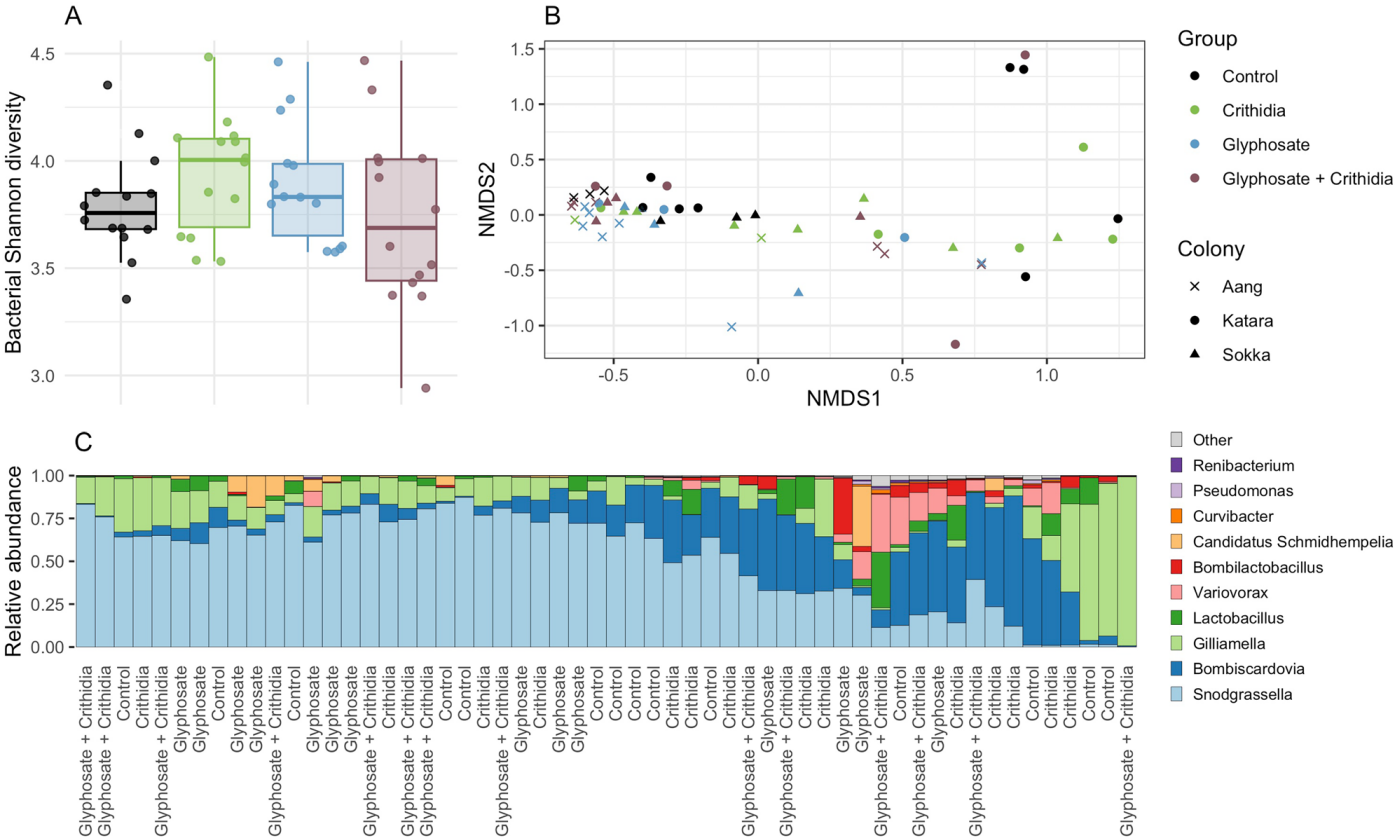
Glyphosate, *Crithidia bombi*, or their combination have limited effects on bacterial composition of the bumblebee microbiome. (**A**) Bacterial diversity was evaluated by 16S rRNA amplicon sequencing. (**B**) Clustering of non-metric multi-dimensional scaling (NMDS) plots of Bray–Curtis dissimilarities. (**C**) Gut microbiome composition at the genus level.

There was no significant effect of any treatment on alpha diversity (DF = 3, F = 1.2, *p* value ANOVA = 0.3) (Fig. 1A). Beta diversity was also not affected by treatment as demonstrated by PERMANOVA analysis (*p* value = 0.07) (Fig. 1B), although gut microbiome profiles did cluster by colony (*p* value = 0.005). We then analysed bacterial taxonomic composition to assess if there were any alterations in microorganisms by exposure to the treatments. Bacterial profiles from the bee gut microbiome (Fig. 1C) consisted of Proteobacteria (*Snodgrassella *spp., *Gilliamella* spp., *Variovorax* spp.), Actinobacteria (*Bombiscardovia* spp.), and Firmicutes (*Lactobacillus* spp.). Overall, no statistically significant differences in bacterial composition were found between treatment groups compared to controls (see the Supplementary Materials for detailed results). These observations suggest that acute exposure to glyphosate, *Crithidia bombi*, or their combination, had no effect on bacterial composition of the bumblebee microbiome.

## Discussion

The effects of glyphosate on the gut microbiome of different animal species, and its health implications is an area of intense investigation and discussion. Here we present the first study to assess the impact of glyphosate alongside a parasite *Crithidia bombi* on the gut microbiome of the buff tailed bumblebee (*Bombus terrestris*). Utilising a fully crossed testing protocol, we found no impact of either glyphosate, *C. bombi*, or their combination on bacterial composition. This result is contrary to observations from studies with honeybees, which has consistently found an impact of glyphosate on gut bacterial composition^26–28,31,59^. In a recent study, chronic exposure to glyphosate and its formulated product Roundup induced a transient reduction in the relative abundance of *Snodgrassella* suggesting that this herbicide can have negative effects on the bumblebee microbiota in some conditions^29^. This difference in findings can be explained by the difference in test species (both honeybee versus bumblebee and *B. impatiens* versus *B. terrestris*), and also by the use of an acute rather than a chronic exposure to the glyphosate. Studies on *B. terrestris* found only limited impacts on the microbiome with chronic exposure, more concordant with our results^45,46^.

Prior research on glyphosate and *C. bombi* found no impacts of either stressor on mortality, longevity, parasite intensity, sucrose consumption and weight change in bumblebees^21^. Neither acute nor an environmentally realistic chronic exposure impacted individual bumblebee health metrices^21^. It is possible that a chronic exposure to glyphosate could lead to an alteration in gut microbiome composition. This is seen in Motta and Moran^29^, and to a highly limited effect in Cullen et al.^45^, but not seen in Tang et al.^46^. Such an exposure has already been shown not to generate a measurable impact on the most basic metrices of bumblebee health, with no observable impacts at field realistic exposures^29,45,46^. Of course, other exposure scenarios do exist, such as a longer-term exposure to a more consistent low dosage, or exposure during a different life history phase, such as the larval phase. As glyphosate is used widely and for a variety of purposes^11^, bees exposure to it can also be very varied^19,60,61^. The impact of different exposure scenarios, or different bee physiology at different life history stages, could lead to different results being observed.

Using a comparable 16S amplicon sequencing method, Motta, Raymann and Moran^26^ found that chronic exposure to glyphosate impacted the gut microbiome of *Apis mellifera*, with a change in relative abundance and composition of gut bacteria. This change to the microbiome did not affect mortality of the bees, which is consistent with regulatory evidence that glyphosate alone is non-lethal to bees^62^. However, in combination with another stressor, the parasite *Serratia marcescens*, glyphosate treated bees died at a significantly higher rate than bees treated with just *S. marcescens*. This was experimentally determined to be due to the changes to the bee’s gut microbiome, causing increased susceptibility to the parasite. This contrasts with our results, where neither an impact on the gut microbiome was detected, nor any form of synergy with the parasite. This indicates that the impacts of glyphosate on bees, and their parasites, is species (and parasite) specific, and not necessarily generalisable across all species of bees. One caveat to this comparison is that *S. marcescens* caused detectable effects in honeybees under the experimental conditions, while *C. bombi* did not have a detectable effect on bumblebees in our experiment. So, while *C. bombi* is a known stressor of bumblebees, the lack of synergism is perhaps unsurprising here.

While no effect was observed within our experimental setting, it would be premature to conclude that glyphosate and *C. bombi* have no effect without considering the different confounding factors that can affect the results of such a gut microbiome study. A strong limitation in our study design is the use of the 16S rRNA sequencing platform, which detects the bacterial genome but does not detect other microorganisms such as fungi and viruses. A recent study investigating the effects of exposure to glyphosate on the rat gut microbiome, found opportunistic fungal grow^63^, possibly caused by a general toxicity on major bacterial commensals, which decreased colonisation resistance. It is possible that an approach that takes fungi and viruses into account may also detect an impact of glyphosate and a parasitic stressor on the bumblebee gut microbiome.

Future studies would benefit from the use of more comprehensive gut microbiome analysis strategies such as shotgun metagenomics. However, these approaches are difficult to conduct on samples with a low microbial biomass as was the case in this study. Extracting DNA from the gut of bumblebees proved challenging. Another possible confounding factor which is known to play a role in the composition of the bumblebee gut microbiome is colony age or the age of individuals within the colony^64^. The age of the individuals was not controlled in our study. However, the colonies arrived young, and the workers were thus likely to be of a similar age. Although colony age/dynamics was not directly controlled in this study, the role of colony allocation was studied and taken into account in the statistical analysis since it was shown that the gut microbiome profiles clustered by colony (Fig. 1B). Further, all colonies were the same age, having been delivered in the same shipment.

The dose of glyphosate used in this experiment, 200 µg, is a very high acute dose. Using the OECD 247^47^ methodology for exposing bees to pesticides, it is difficult to generate a dose higher than 200 µg because of the solubility limit of glyphosate. Accordingly, our exposure represents a worst-case exposure for a single acute ingestion of glyphosate. Given that no effect was detected using this dosage, it is reasonable to assume that at lower doses there would similarly be no effect.

While we used glyphosate as an active ingredient, glyphosate is applied in agriculture in formulations. Here glyphosate is mixed with other compounds called co-formulants, which includes compounds such as surfactants or emulsifiers. These co-formulants are added to the spray mixes in order to increase the stability of the formulation and aid penetration of glyphosate through plant waxy surfaces, promoting its action. These co-formulants can have their own toxic effects in different animals^65,66^ including bees^67–69^. Accordingly, it is important for future research to test whether exposure to commercial glyphosate-based herbicide formulations containing co-formulants and not just glyphosate alone affects the bumblebee gut microbiome (see^29^ and^45^). Recent gut microbiome studies performed in rats^25,70^ and an in vitro simulator of human gut microbiota^63^ both found glyphosate formulations had more effects than glyphosate alone. These observations make it quite possible that the commercial glyphosate formulations will cause more of an impact than the active ingredient glyphosate alone in the bumblebee microbiome. Furthermore, it has been observed that despite little change in gut microbiota composition, the metabolic function of the microbiome can be significantly altered, reflective of oxidative stress following glyphosate herbicide exposure^25^. This again raises the possibility that even though we detected no significant change in bumblebee microbiome composition (Fig. 1), its biochemical function could have been altered, which could be investigated in future studies using a metabolomics approach.

To conclude, these results add novel insights into our understanding of how glyphosate, the world’s most used pesticide, impacts pollinator gut microbiomes. The lack of an effect of glyphosate on bacterial composition seen here in bumblebees is contrary to what has been reproducibly observed in *Apis mellifera*. This indicates that glyphosate can differentially impact the microbiome of different bee species. This is an important distinction as *A. mellifera* is used as a model species to represent pollinators more broadly in a risk assessment context. Accordingly, we advise caution in extrapolating gut microbiome results from *A. mellifera* to other bee species. In addition, future studies need to address the influence of glyphosate commercial formulations on microbiome composition and metabolic activity to obtain a more comprehensive evaluation of effects and their consequences on bee health. As we found no impact of glyphosate on the bumblebee bacterial microbiome, nor any interaction with a common bumblebee parasite, these results do not support calls for changes in the ecotoxicological risk assessment of glyphosate.

## Supplementary Information

Supplementary Information.

## Data Availability

The whole organism response data and the statistical analysis reports from the gut microbiome analysis are available in the supplementary materials. The microbiome dataset generated and analysed during the current study are available in the NCBI repository, https://trace.ncbi.nlm.nih.gov/Traces/?view=study&acc=SRP412675 and BioProject PRJNA911459.
