# Supplementary material for: No impacts of glyphosate or Crithidia bombi, or their combination, on the bumblebee microbiome
Source: Sci Rep. 2023 Jun 2;13:8949. doi: 10.1038/s41598-023-35304-3 (PMC10238469; doi:10.1038/s41598-023-35304-3)
Supplement: Supplementary file 1 — Supplementary Information. [file 41598_2023_35304_MOESM1_ESM.docx]

**Supplementary Materials: No impacts of glyphosate or *Crithidia bombi*, or their combination, on the bumblebee microbiome.**

**Edward A. Straw^1,3*^, Robin Mesnage^2,4*^, Mark J. F. Brown^3^, and Michael N. Antoniou^4^**

^1^ Department of Botany, Trinity College Dublin, Dublin, Ireland.

^2^ Buchinger Wilhelmi Clinic, Wilhelmi-Beck-Straße 27, 88662 Überlingen, Germany

^3^ Department of Biological Sciences, School of Life Sciences and the Environment, Centre for Ecology, Evolution & Behaviour, Royal Holloway University of London, Egham, UK.

^4^ Gene Expression and Therapy Group, King's College London, Faculty of Life Sciences & Medicine, Department of Medical and Molecular Genetics, Guy's Hospital, London, SE1 9RT, UK.

* Corresponding authors, contributed equally to the work

**Results**

*Whole organism results*

*Sucrose consumption*

There was no impact of colony on sucrose consumption (Katara: parameter estimate (PE) = -0.04, 95% Confidence Intervals (CI) [-0.10 to 0.03]; Sokka: PE = -0.02, 95% CI [-0.08 to 0.04]).

*Weight change*

There was no impact of colony on weight change (Katara: parameter estimate (PE) = -0.00, 95% Confidence Intervals (CI) [-0.07 to 0.07]; Sokka: PE = -0.01, 95% CI [-0.06 to 0.04]).

*Gut microbiome composition*

See Supplementary Table 1.

**Supplementary Table 1.** Differences in the composition of the microbiota. Statistical models were established with MaAsLin2. The model coefficient value (effect size) and the standard error from the model are reported in comparison to the control group along the p-values and its False Discovery Rate (FDR).

| Feature | Value | coef | stderr | pval | FDR |
| --- | --- | --- | --- | --- | --- |
| Candidatus.Schmidhempelia | Glyphosate | 0.119 | 0.044 | 0.010 | 0.807 |
| Snodgrassella | Crithidia | -0.130 | 0.126 | 0.307 | 0.986 |
| Snodgrassella | Glyphosate | 0.093 | 0.126 | 0.462 | 0.986 |
| Snodgrassella | Glyphosate + Crithidia | 0.033 | 0.126 | 0.797 | 0.986 |
| Bombiscardovia | Crithidia | 0.202 | 0.093 | 0.035 | 0.986 |
| Bombiscardovia | Glyphosate | -0.033 | 0.093 | 0.725 | 0.986 |
| Bombiscardovia | Glyphosate + Crithidia | -0.037 | 0.093 | 0.697 | 0.986 |
| Gilliamella | Crithidia | -0.073 | 0.103 | 0.483 | 0.986 |
| Gilliamella | Glyphosate | -0.117 | 0.103 | 0.258 | 0.986 |
| Gilliamella | Glyphosate + Crithidia | -0.051 | 0.103 | 0.624 | 0.986 |
| Lactobacillus | Crithidia | 0.059 | 0.055 | 0.292 | 0.986 |
| Lactobacillus | Glyphosate | 0.008 | 0.055 | 0.888 | 0.986 |
| Lactobacillus | Glyphosate + Crithidia | 0.036 | 0.055 | 0.520 | 0.986 |
| Variovorax | Crithidia | 0.025 | 0.059 | 0.665 | 0.986 |
| Variovorax | Glyphosate | 0.012 | 0.059 | 0.841 | 0.986 |
| Variovorax | Glyphosate + Crithidia | 0.018 | 0.059 | 0.759 | 0.986 |
| Bombilactobacillus | Crithidia | 0.015 | 0.042 | 0.722 | 0.986 |
| Bombilactobacillus | Glyphosate | 0.032 | 0.042 | 0.453 | 0.986 |
| Bombilactobacillus | Glyphosate + Crithidia | -0.022 | 0.042 | 0.613 | 0.986 |
| Candidatus.Schmidhempelia | Crithidia | 0.013 | 0.044 | 0.766 | 0.986 |
| Candidatus.Schmidhempelia | Glyphosate + Crithidia | 0.012 | 0.044 | 0.782 | 0.986 |
| Curvibacter | Crithidia | 0.006 | 0.014 | 0.683 | 0.986 |
| Curvibacter | Glyphosate | 0.001 | 0.014 | 0.929 | 0.986 |
| Curvibacter | Glyphosate + Crithidia | 0.001 | 0.014 | 0.950 | 0.986 |
| Luteibacter | Crithidia | 0.005 | 0.010 | 0.651 | 0.986 |
| Luteibacter | Glyphosate | 0.000 | 0.010 | 0.974 | 0.986 |
| Luteibacter | Glyphosate + Crithidia | 0.003 | 0.010 | 0.742 | 0.986 |
| Renibacterium | Crithidia | 0.002 | 0.011 | 0.849 | 0.986 |
| Renibacterium | Glyphosate | 0.003 | 0.011 | 0.789 | 0.986 |
| Renibacterium | Glyphosate + Crithidia | 0.001 | 0.011 | 0.950 | 0.986 |
| Leifsonia | Crithidia | 0.005 | 0.009 | 0.558 | 0.986 |
| Leifsonia | Glyphosate | 0.003 | 0.009 | 0.758 | 0.986 |
| Leifsonia | Glyphosate + Crithidia | 0.003 | 0.009 | 0.774 | 0.986 |
| Bradyrhizobium | Crithidia | 0.011 | 0.007 | 0.132 | 0.986 |
| Bradyrhizobium | Glyphosate | 0.002 | 0.007 | 0.748 | 0.986 |
| Bradyrhizobium | Glyphosate + Crithidia | 0.009 | 0.007 | 0.239 | 0.986 |
| Pseudomonas | Crithidia | 0.001 | 0.013 | 0.968 | 0.986 |
| Pseudomonas | Glyphosate | -0.006 | 0.013 | 0.624 | 0.986 |
| Pseudomonas | Glyphosate + Crithidia | -0.007 | 0.013 | 0.576 | 0.986 |
| Sphingobium | Crithidia | 0.002 | 0.007 | 0.800 | 0.986 |
| Sphingobium | Glyphosate | -0.001 | 0.007 | 0.854 | 0.986 |
| Sphingobium | Glyphosate + Crithidia | 0.004 | 0.007 | 0.560 | 0.986 |
| Pelomonas | Crithidia | 0.004 | 0.007 | 0.622 | 0.986 |
| Pelomonas | Glyphosate | 0.003 | 0.007 | 0.676 | 0.986 |
| Pelomonas | Glyphosate + Crithidia | 0.004 | 0.007 | 0.568 | 0.986 |
| Sphingomonas | Crithidia | 0.009 | 0.007 | 0.185 | 0.986 |
| Sphingomonas | Glyphosate | 0.001 | 0.007 | 0.888 | 0.986 |
| Sphingomonas | Glyphosate + Crithidia | 0.008 | 0.007 | 0.268 | 0.986 |
| Azospirillum | Crithidia | 0.003 | 0.006 | 0.603 | 0.986 |
| Azospirillum | Glyphosate | 0.002 | 0.006 | 0.785 | 0.986 |
| Azospirillum | Glyphosate + Crithidia | 0.003 | 0.006 | 0.608 | 0.986 |
| Escherichia.Shigella | Crithidia | 0.007 | 0.006 | 0.214 | 0.986 |
| Escherichia.Shigella | Glyphosate | 0.000 | 0.006 | 0.971 | 0.986 |
| Escherichia.Shigella | Glyphosate + Crithidia | 0.001 | 0.006 | 0.828 | 0.986 |
| Paracoccus | Glyphosate | 0.002 | 0.005 | 0.664 | 0.986 |
| Paracoccus | Glyphosate + Crithidia | -0.001 | 0.005 | 0.844 | 0.986 |
| Corynebacterium | Crithidia | -0.001 | 0.004 | 0.855 | 0.986 |
| Corynebacterium | Glyphosate | -0.002 | 0.004 | 0.628 | 0.986 |
| Corynebacterium | Glyphosate + Crithidia | 0.002 | 0.004 | 0.621 | 0.986 |
| Burkholderia.Caballeronia | Crithidia | -0.002 | 0.003 | 0.530 | 0.986 |
| Burkholderia.Caballeronia | Glyphosate | -0.002 | 0.003 | 0.580 | 0.986 |
| Burkholderia.Caballeronia | Glyphosate + Crithidia | -0.004 | 0.003 | 0.134 | 0.986 |
| Aquabacterium | Crithidia | -0.002 | 0.004 | 0.553 | 0.986 |
| Aquabacterium | Glyphosate | 0.000 | 0.004 | 0.967 | 0.986 |
| Aquabacterium | Glyphosate + Crithidia | -0.003 | 0.004 | 0.400 | 0.986 |
| Massilia | Crithidia | 0.001 | 0.005 | 0.829 | 0.986 |
| Massilia | Glyphosate | -0.001 | 0.005 | 0.876 | 0.986 |
| Massilia | Glyphosate + Crithidia | 0.001 | 0.005 | 0.910 | 0.986 |
| Staphylococcus | Crithidia | 0.003 | 0.004 | 0.520 | 0.986 |
| Staphylococcus | Glyphosate | -0.002 | 0.004 | 0.621 | 0.986 |
| Staphylococcus | Glyphosate + Crithidia | 0.001 | 0.004 | 0.822 | 0.986 |
| Acinetobacter | Crithidia | -0.005 | 0.006 | 0.393 | 0.986 |
| Acinetobacter | Glyphosate | -0.005 | 0.006 | 0.390 | 0.986 |
| Acinetobacter | Glyphosate + Crithidia | -0.004 | 0.006 | 0.453 | 0.986 |
| Methylobacterium | Crithidia | 0.003 | 0.003 | 0.374 | 0.986 |
| Methylobacterium | Glyphosate | 0.002 | 0.003 | 0.586 | 0.986 |
| Methylobacterium | Glyphosate + Crithidia | 0.002 | 0.003 | 0.541 | 0.986 |
| Stenoxybacter | Crithidia | -0.002 | 0.003 | 0.422 | 0.986 |
| Stenoxybacter | Glyphosate | 0.003 | 0.003 | 0.248 | 0.986 |
| Stenoxybacter | Glyphosate + Crithidia | 0.001 | 0.003 | 0.859 | 0.986 |
| Paracoccus | Crithidia | 0.0001 | 0.005 | 0.996 | 0.996 |
